# Network Pharmacology and Molecular Docking for Natural Product-Based Therapies in Gallbladder and Biliary Tract Cancers: Workflows, Pitfalls, and Translational Opportunities

**DOI:** 10.7759/cureus.110851

**Published:** 2026-06-14

**Authors:** Sushant S Das, Rajni Rathore, Harsimranjit Singh, Shalika Sharma, M Ramkumar

**Affiliations:** 1 Anatomy, All India Institute of Medical Sciences, Jammu, IND; 2 Pharmacology and Clinical Skills, Medical University of the Americas, Nevis, KNA

**Keywords:** biliary tract cancers, gallbladder cancer, molecular docking, natural products, network pharmacology, translational pharmacology

## Abstract

Gallbladder cancer (GBC) and other biliary tract cancers (BTCs) are aggressive hepatobiliary malignancies with limited pharmacological treatment options and a poor prognosis. Even with platinum-gemcitabine regimens and selected targeted or immunotherapies, most patients experience only transient benefits, highlighting the need for new mechanistic approaches. Natural products, ranging from purified phytochemicals to complex traditional formulations, act on several key processes involved in gallbladder carcinogenesis, including chronic inflammation, epithelial-mesenchymal transition, apoptosis, and DNA damage responses. These multi-target effects are difficult to capture using reductionist approaches that focus on single receptors or pathways. Network pharmacology and molecular docking have therefore emerged as valuable in silico tools for understanding this complexity. By integrating compound-target predictions, protein-protein interaction networks, and pathway enrichment analyses with structural models of ligand-target binding, these approaches can generate pharmacological hypotheses, prioritize targets for validation, and suggest rational combinations with standard systemic therapies. However, their application to gallbladder and biliary tract cancers remains limited and methodologically heterogeneous. In this narrative review, we summarize current preclinical evidence, outline standard workflows, critically examine common pitfalls, and propose a best-practice workflow and reporting checklist. We also discuss how in silico methods can be integrated into experimental and clinical pharmacology to support the mechanism-driven development of natural product-based therapies in hepatobiliary oncology.

## Introduction and background

Gallbladder cancer (GBC) and related biliary tract cancers (BTCs) remain among the most lethal gastrointestinal malignancies [1]. Although they are often grouped together clinically, GBC differs biologically from intrahepatic and extrahepatic cholangiocarcinomas, with a stronger association with chronic inflammation and cholelithiasis, as well as a distinct pattern of driver alterations, including recurrent ELF3 and ERBB2 changes in GBC cohorts [2-4]. These tumors show marked geographic variation, with particularly high incidence and mortality reported in parts of South Asia (including northern India), Eastern Europe, and Latin America, underscoring their disproportionate regional public health impact [1,5].

Most patients present with unresectable or metastatic disease, and recurrence is common even among those who undergo surgery [6]. Systemic therapy continues to rely primarily on gemcitabine-platinum-based chemotherapy. However, the current first-line standard increasingly incorporates chemoimmunotherapy, with durvalumab or pembrolizumab added to the gemcitabine-cisplatin backbone, based on the results of the TOPAZ-1 and KEYNOTE-966 phase III trials [7-9]. Despite these advances, most patients derive only modest and often transient benefit. Consequently, there remains a clear need for new therapeutic targets, novel mechanisms of action, and rational adjunctive strategies that can improve survival without adding unacceptable toxicity [10,11].

Natural products have long contributed to the oncology pharmacopoeia, ranging from classical cytotoxic agents such as vinca alkaloids and taxanes to the growing interest in phytochemicals and complex traditional formulations [12,13]. In gallbladder and biliary tract cancers, preclinical studies suggest that several plant-derived compounds and natural mixtures can modulate key hallmarks of cancer, including proliferation, apoptosis, angiogenesis, inflammation, and immune responses [14]. These agents typically act through multiple molecular targets and signaling pathways, offering the theoretical advantage of network-level modulation of carcinogenic processes [14,15]. In particular, a single natural extract may contain dozens of phytochemicals that act in parallel on multiple interconnected signaling nodes, meaning that its pharmacology cannot be adequately explained by a single drug-receptor interaction.

Capturing and rationalizing these pleiotropic pharmacological effects is challenging within traditional one-target-one-drug frameworks [16]. Network pharmacology and molecular docking have therefore gained traction as complementary in silico approaches that map compound-target-disease relationships and generate structural hypotheses for ligand-protein interactions [17,18]. Network pharmacology is a systems biology-oriented approach that examines how multiple compounds interact with interconnected molecular targets and pathways simultaneously, rather than focusing on a single drug-receptor pair [19]. Molecular docking is a computational technique used to predict how small molecules bind to target proteins and to generate structural models of these putative ligand-protein interactions [20]. Over the past decade, these methods have been widely applied to traditional medicine formulations and natural products across multiple disease areas, including cancer [21,22]. However, their application to gallbladder and biliary tract cancers remains limited and methodologically heterogeneous.

This review aims to bridge pharmacology and systems-based approaches in this field. We first outline the pharmacological challenges and unmet needs in gallbladder and biliary tract cancers and summarize the current preclinical evidence for natural product-based interventions. We then introduce the key concepts and standard workflows of network pharmacology and molecular docking and critically evaluate their application to natural products in oncology. Building on this analysis, as well as our own experience with network-based studies, we propose a best-practice pharmacological workflow and a reporting checklist that we believe will enhance rigor, reproducibility, and translational relevance. Finally, we discuss how in silico pharmacology can be more effectively integrated with experimental pharmacology and early-phase clinical research to support the mechanism-driven development of natural product-based therapies for gallbladder and biliary tract cancers.

## Review

Review methodology

Although this is a narrative rather than a systematic review, a comprehensive literature search was conducted in PubMed, Ovid Discovery, Scopus, and Web of Science from January 2010 to April 2026. The final search was performed on April 10, 2026. The objective was to identify studies that applied network pharmacology and/or molecular docking to natural products or traditional formulations in GBC, BTCs, or closely related hepatobiliary malignancies. In PubMed, the following search string was used: ("Gallbladder Neoplasms"[MeSH] OR "Bile Duct Neoplasms"[MeSH] OR gallbladder cancer OR biliary tract cancer OR cholangiocarcinoma OR hepatobiliary cancer) AND (natural products OR phytochemicals OR herbal OR "traditional medicine") AND ("Network Pharmacology"[MeSH] OR molecular docking OR "systems pharmacology" OR "target prediction" OR "bioinformatics approach"). Equivalent Boolean search strategies and database-specific subject headings were adapted for Ovid Discovery, Scopus, and Web of Science. These searches were supplemented by manual screening of the reference lists of included articles. The inclusion criteria comprised peer-reviewed, English-language original research articles that (1) evaluated one or more natural products, (2) incorporated a network-based analysis (compound-target-disease networks, protein-protein interaction networks, or pathway enrichment analyses) and/or molecular docking, and (3) reported findings relevant to GBC, other BTCs, or hepatobiliary malignancies that share mechanistic pathways implicated in GBC/BTC. Studies were excluded if they were purely in silico investigations that did not clearly specify the disease or clinical context, focused on non-malignant hepatobiliary conditions, or were non-original publications (e.g., editorials, letters, or conference abstracts).

The search yielded 228 records after duplicate removal. Following title and abstract screening, 123 records were excluded, and 105 articles underwent full-text assessment. Of these, 83 papers were ultimately included in the narrative synthesis. Two independent reviewers screened titles, abstracts, and full texts, with disagreements resolved by a third reviewer. Given the heterogeneity of methodologies and the limited number of studies specifically focused on GBC and BTCs, a narrative synthesis was considered more appropriate than a formal systematic review with meta-analysis. Accordingly, no formal risk-of-bias assessment tool was applied to individual studies. Instead, emphasis was placed on identifying recurrent methodological strengths and limitations across the literature, and all in silico findings were interpreted as hypothesis-generating rather than confirmatory.

GBCs and BTCs: pharmacological challenges and unmet needs

GBC forms part of the spectrum of BTCs, which also includes intrahepatic and extrahepatic cholangiocarcinomas [22]. Although these entities differ in anatomical site, epidemiology, and risk factors, they share a poor overall prognosis and substantial therapeutic challenges [22,23]. Most patients present with locally advanced or metastatic disease because of nonspecific early symptoms and the lack of effective screening strategies [24,25]. Even after curative resection, recurrence is common, reflecting both aggressive tumor biology and the limitations of current adjuvant therapies [26].

Systemic treatment for advanced BTCs has, until recently, been dominated by cytotoxic chemotherapy [27]. Gemcitabine-platinum combinations remain the backbone of first-line therapy and provide only modest improvements in survival and symptom control [28]. Incremental gains have been achieved through optimization of dosing schedules and the exploration of doublet and triplet regimens. However, primary and acquired resistance, cumulative toxicity, and narrow therapeutic indices continue to pose major challenges [11,29]. More recently, targeted therapies directed against specific molecular alterations, such as FGFR2 fusions and IDH1 mutations in cholangiocarcinoma, as well as HER2-directed therapies in selected BTC subsets, have improved outcomes in molecularly characterized patient populations. Similarly, chemoimmunotherapy combinations, including durvalumab-gemcitabine-cisplatin and pembrolizumab-gemcitabine-cisplatin, have demonstrated clinical benefit in biomarker-selected or broader BTC populations [8,30,31]. However, these advances currently benefit only a minority of patients, and actionable genomic alterations appear less frequent and less well characterized in GBC than in other BTC subtypes [27,32]. In addition, access to biomarker-driven therapies remains uneven in many high-incidence regions [22].

The tumor microenvironment of BTCs poses additional pharmacological challenges [33]. A dense desmoplastic stroma, aberrant vasculature, and an immunosuppressive milieu can limit drug penetration, promote therapeutic resistance, and dampen antitumor immune responses [33,34]. Coexisting liver dysfunction and biliary obstruction further narrow the therapeutic window by increasing the risk of systemic toxicity and drug-drug interactions [35,36]. Together, these factors help explain the limited durability of the benefit associated with current systemic therapies and highlight the need for strategies that move beyond single-target paradigms [22].

Mechanistically, gallbladder and biliary tract carcinogenesis involves the convergent dysregulation of multiple signaling pathways. These include chronic inflammatory signaling, genomic instability, dysregulated apoptosis and cell-cycle control, altered metabolism, and crosstalk with the tumor microenvironment [23,37]. Targeting any single node in isolation may therefore yield only partial and transient disease control [11]. This multifactorial landscape provides a strong rationale for pharmacological strategies that can simultaneously modulate multiple pathways involved in tumor initiation, progression, and treatment resistance [11]. Natural products and complex traditional formulations, with their inherent multi-component and multi-target properties, represent attractive candidates in this regard. However, their network-level pharmacology must be rigorously characterized and aligned with translational objectives [38,39].

Natural products in GBC/BTC: preclinical pharmacology

Mechanistic Themes and Preclinical Evidence

A growing body of preclinical evidence suggests that selected natural products can modulate key molecular and cellular processes involved in GBC and BTC [14]. Much of this work has focused on isolated phytochemicals derived from medicinal plants with an established track record in oncology, including curcumin, resveratrol, baicalein, and related polyphenols [14,40]. In GBC cell lines and experimental models, these agents have been reported to inhibit proliferation, induce cell-cycle arrest, trigger intrinsic and extrinsic apoptosis, modulate oxidative stress, and interfere with signaling pathways involved in inflammation and epithelial-mesenchymal transition (EMT) [14,41]. In some cases, natural products have also been shown to impair migratory and invasive behavior or enhance the sensitivity of tumor cells to standard chemotherapeutic agents [41,42].

Systematic assessments of natural products in GBC further support these mechanistic themes. Across diverse compounds and extracts, recurrent targets include regulators of NF-κB and STAT signaling, pro- and anti-apoptotic proteins, cell-cycle checkpoints, and components of the PI3K-AKT and MAPK pathways. In vivo, phytochemicals and plant extracts have demonstrated tumor growth inhibition and, in some models, reduced metastatic burden. These effects are often accompanied by changes in angiogenic markers, inflammatory cytokines, and indices of oxidative stress. Although many of these studies are exploratory and heterogeneous in design, the collective evidence suggests that natural products can engage multiple functionally convergent pathways that are directly relevant to gallbladder and biliary tract cancers [14,15].

In GBC and other BTCs, integrated genomic and transcriptomic analyses have identified alterations in ELF3, ERBB2, PIK3CA, and TP53, together with chronic NF-κB activation, PI3K-AKT signaling, and EMT-related transcription factors, as recurrent drivers of tumor behavior. Our synthesis indicates that many natural products evaluated in GBC and BTC cell lines converge on these same biological axes, modulating NF-κB, STAT3, and PI3K-AKT signaling, as well as oxidative stress responses and EMT markers. These observations suggest that disease-specific network models should prioritize these pathways when interpreting enrichment analyses and proposing mechanisms of action [3,14,43].

Among complex natural preparations, mineral-rich organic matrices such as Shilajit have attracted attention as potential anticancer adjuvants [44]. These materials contain mixtures of fulvic acids, dibenzo-α-pyrones, and other low-molecular-weight constituents [45]. Preclinical studies in non-hepatobiliary cancer models have suggested antiproliferative, pro-apoptotic, and antioxidant effects, as well as modulation of mitochondrial function and attenuation of inflammatory mediators [44]. In some experimental systems, Shilajit-like preparations have also enhanced the antitumor activity of conventional anticancer drugs or reduced treatment-related toxicity [46]. However, evidence specific to GBC and BTCs remains limited, and extrapolation from other tumor types should be approached with caution [44]. In this context, we cite Shilajit primarily as an example of a complex multi-component matrix for which disease-specific network pharmacology could, in principle, facilitate constituent standardization and map predicted targets onto GBC/BTC-relevant biological networks. Such networks should be enriched for the GBC/BTC driver genes and signaling pathways outlined above, rather than being interpreted as evidence of established hepatobiliary pharmacology.

Translational Challenges and Rationale for Network Pharmacology

Several issues complicate the interpretation and translation of these findings. First, the quality and design of preclinical studies vary widely, with differences in cell lines, animal models, dosing regimens, and study endpoints [47]. Second, many natural products are tested at concentrations that may not be achievable in human tissues, while pharmacokinetic data remain limited for numerous compounds and preparations [48]. Third, complex natural matrices present challenges related to standardization, batch-to-batch variability, and potential contamination, all of which can confound efficacy and safety assessments [49]. Collectively, these factors highlight the need for more rigorous experimental pharmacology and for integrative frameworks capable of prioritizing the most promising targets, pathways, and compound combinations for further investigation.

Against this backdrop, network pharmacology and molecular docking provide attractive complements to traditional preclinical approaches [50]. By systematically mapping the putative targets and pathways engaged by multiple natural product constituents, these in silico methods can help rationalize the pleiotropic effects observed in vitro and in vivo [51]. They can also explore structural interactions with key proteins, thereby providing additional mechanistic insight [17]. Furthermore, they may identify mechanistically informed combinations with standard chemotherapy and highlight potential off-target liabilities [51]. For GBC and BTCs in particular, such approaches offer a means of organizing the diverse effects of natural products around disease-defining pathways and molecular alterations, rather than treating them as generic anticancer agents. However, to realize this potential in GBC and BTCs, in silico analyses must remain closely aligned with, and continuously informed by, high-quality experimental pharmacology.

Network pharmacology and molecular docking: concepts and workflows

Network pharmacology extends classical pharmacology by considering drugs, targets, and diseases as elements of interconnected biological networks rather than isolated one-to-one relationships [52]. This perspective is particularly relevant to natural products, which often comprise multiple bioactive constituents that act on partially overlapping sets of proteins and pathways [53,54]. Instead of asking whether a single compound modulates a single receptor, network pharmacology examines how a constellation of compounds perturbs a disease-relevant network and how those perturbations relate to observed pharmacological phenotypes [52].

A standard network pharmacology workflow begins with a rigorous definition of the pharmacological question and intervention [55]. Investigators must first be clear about their objective, whether it is to elucidate mechanisms underlying observed anticancer effects, identify potential synergistic targets for existing drugs, or prioritize candidate compounds for further development in GBC and BTCs [52,56]. The natural product under investigation, whether a single phytochemical, a defined mixture, or a complex formulation, should then be characterized as comprehensively as possible. This characterization should include its composition, source, method of preparation, and any available pharmacokinetic or toxicological data [55,56].

The next step is the systematic collection of compounds and targets. Candidate molecules are identified through phytochemical profiling or retrieved from well-curated databases. Putative protein targets are then predicted or obtained using ligand-based, structure-based, and knowledge-based approaches [50,57,58]. In parallel, disease-associated genes and proteins are compiled from curated resources and, where possible, from GBC- and BTC-specific transcriptomic or genomic datasets. Where available, proteomic, metabolomic, and single-cell sequencing data can further refine disease gene sets and network topology by capturing post-transcriptional regulation, metabolic reprogramming, and intratumoral heterogeneity. We recommend that future GBC/BTC network studies explicitly report which omics layers were integrated and how discrepancies between them were addressed. The overlap and union of compound-related and disease-related targets are then used to construct compound-target and protein-protein interaction networks [59-61]. Network topology metrics, such as node degree, betweenness centrality, and clustering coefficients, help identify hubs and modules that may represent pharmacologically important nodes or subnetworks [57,60].

When building GBC/BTC-specific networks, investigators should explicitly incorporate gallbladder-focused gene sets from recent omics studies and annotate how natural product-target interactions intersect with known mutational and pathway landscapes, including recurrent alterations in ELF3, ERBB2, PIK3CA, TP53, and related signaling axes [3,62-64].

Functional enrichment analysis provides a bridge between network structure and biological interpretation [65]. Gene Ontology categories and pathway resources (e.g., KEGG, Reactome, and related databases) are used to identify overrepresented biological processes and signaling pathways among the targets. By highlighting pathways involved in apoptosis, cell-cycle regulation, inflammation, angiogenesis, and epithelial-mesenchymal transition, enrichment analyses can generate testable hypotheses regarding the systems-level mechanisms of natural products [66,67]. Robust workflows should predefine the statistical background, apply appropriate multiple-testing corrections, and report effect sizes alongside p-values. Transparent reporting of software tools, database versions, and analytical thresholds is also essential [66,68,69]. In the context of GBC/BTC, enrichment results should be interpreted in light of established disease biology, for example, by assessing whether natural product-modulated networks reinforce or challenge current models centered on NF-κB, STAT3, PI3K-AKT signaling, bile-related inflammation, and EMT-associated transcription factors [14,43].

Molecular docking complements network pharmacology by providing a structural perspective on compound-target relationships [70]. Typically, selected compounds, often those linked to network hubs or key pathways, are docked into the binding sites of prioritized protein targets using curated three-dimensional structures [70,71]. Reliable predictions depend on careful preparation of protein and ligand structures, appropriate selection of docking algorithms, and validation against known ligands or other controls [72,73]. Estimates of binding affinity and interaction patterns are best interpreted comparatively rather than as precise absolute measures and are primarily used to rank or prioritize candidate interactions suggested by network analyses [70,74].

In more advanced implementations, these core steps may be augmented by additional analytical layers. These include molecular dynamics simulations to refine docking poses and binding energetics, in silico ADMET profiling to anticipate pharmacokinetic and safety issues, and integration with transcriptomic or clinical outcome data [75-77]. Crucially, however, even the most sophisticated network and docking analyses should be embedded within an iterative pharmacological framework. In such a framework, in silico outputs guide the design of in vitro and in vivo experiments, experimental findings feed back to refine networks and models, and emerging clinical observations ultimately test the relevance of proposed mechanisms in gallbladder and biliary tract cancers [75]. For example, docking-prioritized interactions involving ERBB2, PI3K-AKT pathway components, or EMT regulators can be evaluated in GBC/BTC models that measure apoptosis, invasion, chemosensitization, and resistance pathways, thereby closing the loop between disease-specific omics data, network predictions, and functional validation.

Current network pharmacology applications to natural products in cancer, with emphasis on GBC/BTC

In cancer research, network pharmacology and molecular docking have been widely applied to deconvolute the mechanisms of traditional herbal formulations, isolated phytochemicals, and dietary compounds. Studies typically report networks linking dozens of compounds to hundreds of targets, with enrichment in canonical cancer-related pathways and apoptotic signaling [78-80]. However, these enriched pathways often reflect broad cancer biology and database popularity rather than rigorous disease-specific modelling. Indeed, many such studies exemplify the low specificity and overgeneralization biases that we highlight as key methodological pitfalls in this review. Docking analyses are then used to illustrate potential interactions between selected compounds and high-degree or otherwise prioritized targets identified through network analysis [81,82]. Although only a small subset of these studies focuses directly on GBC/BTC, the pathways they highlight overlap strikingly with those identified in gallbladder-focused omics analyses, reinforcing the relevance of PI3K-AKT, NF-κB, STAT3, and EMT-related nodes as key coordinators of natural product activity across this disease spectrum [3,43,62].

For example, Yinchenhao decoction has been investigated in cholangiocarcinoma using a combined network pharmacology, molecular docking, and in vivo validation approach. Investigators identified 32 putative active components and 209 targets, with key hubs including AKT1, IL6, MAPK1, TP53, and VEGFA enriched in MAPK and PI3K-AKT signaling pathways. Subsequent experimental studies demonstrated that the decoction reduced tumor growth and modulated apoptosis-related proteins in preclinical models [83]. Although methodologically stronger than many purely in silico studies, this work still relies on broad cancer pathway enrichment and highlights the importance of explicitly incorporating GBC/BTC-specific gene sets and pharmacokinetic considerations into future network pharmacology workflows.

Despite this apparent richness, the pharmacological insights derived from many such studies are often constrained by methodological variability and incomplete reporting. Different groups employ diverse databases and analytical tools, frequently with limited justification or transparency regarding parameter choices, target inclusion criteria, and quality-control procedures [50,84]. Network analyses may emphasize visual complexity over quantitative rigor [18]. Functional enrichment results are sometimes overinterpreted without appropriate multiple-testing correction or consideration of effect sizes [66,69]. Molecular docking is frequently performed against a small number of arbitrarily selected targets using simplified protocols and without appropriate positive or negative controls. Yet the resulting scores are often interpreted as strong evidence of binding and mechanism [85-87].

These limitations are amplified when studying less well-characterized malignancies such as GBC and BTCs, where disease-specific omics data and experimentally validated targets remain comparatively scarce [88,89]. As a result, most network pharmacology and molecular docking studies of natural products in these cancers generate intriguing hypotheses but fall short of providing robust, translationally relevant pharmacological frameworks. Addressing this gap will require future GBC/BTC-focused studies to explicitly integrate gallbladder-specific gene sets, annotate natural product-target interactions against recurrent driver alterations (such as ELF3, ERBB2, PIK3CA, and TP53), and predefine experimental readouts that test whether predicted network and docking nodes genuinely modulate these disease-defining pathways. Progress will also require greater attention to methodological quality, transparent reporting, and systematic integration of in silico findings with experimental pharmacology and, ultimately, clinical observations [89-92].

Methodological pitfalls, biases, and limits to pharmacological interpretation

Several recurrent pitfalls limit the extent to which network pharmacology and molecular docking studies of natural products can be interpreted as robust pharmacological evidence.

First, target prediction and disease gene selection are major sources of bias. The tools used to infer compound targets often rely on similarity to known ligands or on limited structural data. As a result, they tend to favor well-studied proteins and pathways while overlooking less well-characterized but potentially important targets [93-95]. Disease gene sets assembled from heterogeneous databases or broad search terms may also include genes with only tenuous links to GBC or BTCs. Without careful curation and transparent justification of inclusion criteria, the resulting networks risk reflecting database popularity rather than genuine disease biology [3,96,97].

Second, compound selection and representation are often suboptimal. Complex preparations, including mineral-rich exudates and multi-herb formulations, are sometimes reduced to a small set of “representative” compounds selected from databases rather than identified through rigorous chemical profiling [98,99]. Physicochemical and drug-likeness filters may then be applied without considering formulation context, route of administration, or known metabolites. Such practices can distort the true pharmacological space of an intervention, bias network construction, and consequently mislead downstream analyses [100,101].

Third, network and enrichment analyses are vulnerable to overinterpretation. Topological metrics such as degree and betweenness centrality are sometimes treated as mechanistic indicators without adequate consideration of the quality and completeness of the underlying biological data [102,103]. Enrichment analyses conducted across multiple pathway databases without stringent multiple-testing correction can generate statistically significant but biologically trivial associations. Reporting only pathway names, without presenting the underlying gene-level evidence or effect sizes, further limits meaningful pharmacological interpretation [66,104,105].

Fourth, docking protocols are frequently simplified and inadequately benchmarked [106]. Protein structures may be selected for convenience rather than biological relevance, and binding sites may be defined using default settings rather than experimental evidence [107]. In the absence of validation against known ligands and careful optimization of docking parameters, predicted binding scores and poses should be interpreted cautiously [108]. Nevertheless, they are often treated as quasi-quantitative evidence of binding affinity and mechanism, sometimes in the absence of any experimental corroboration [86].

Finally, the connection to experimental and clinical pharmacology remains weak in many studies. In silico findings are rarely integrated with existing in vitro and in vivo evidence derived from the same compounds or disease models. Important considerations, including achievable drug concentrations, pharmacokinetics, toxicity, and drug-drug interactions, are also seldom addressed. This disconnect limits the utility of network pharmacology and molecular docking outputs for guiding mechanistic investigations, optimizing dosing and scheduling strategies, and informing the design of early-phase clinical trials in GBC and BTCs [89,109,110].

Best‑practice pharmacological workflow and reporting checklist

**Figure 1:**
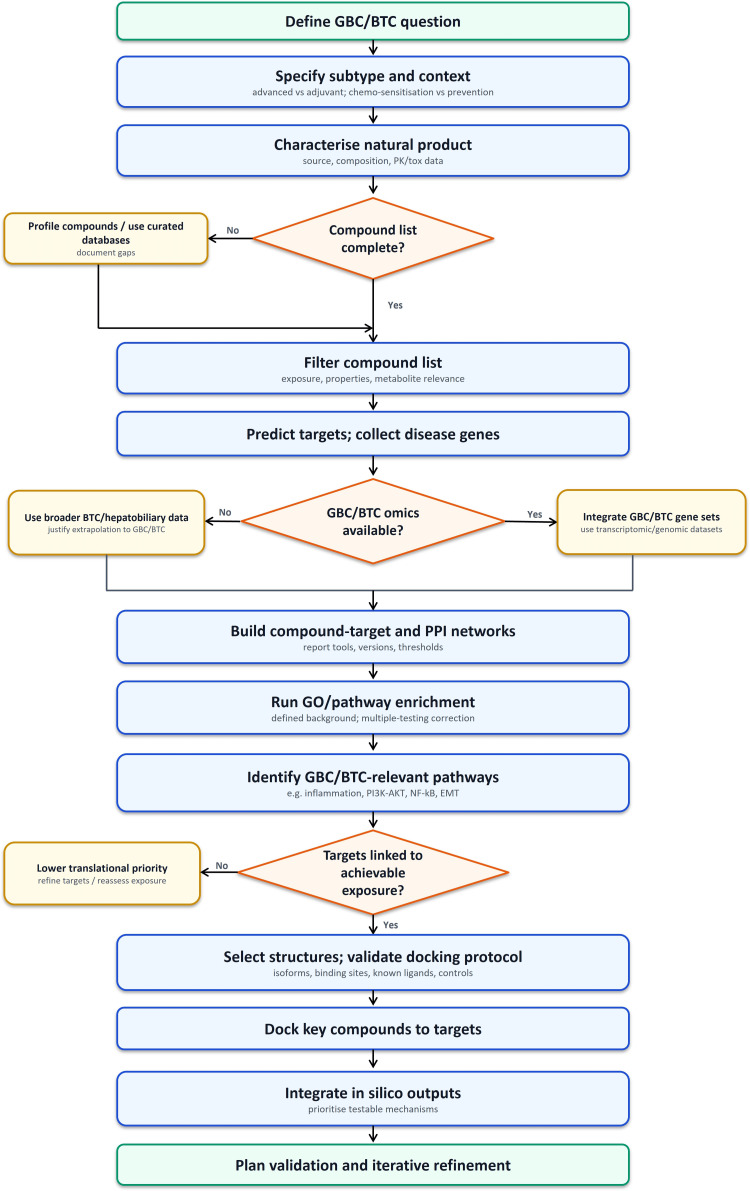
Methodological decision tree highlighting key decision points in network pharmacology and molecular docking workflows for natural product-based studies in gallbladder and biliary tract cancers, including compound selection, disease-specific target definition, in silico prioritization, and planning of experimental validation. BTC: biliary tract cancer, EMT: epithelial-mesenchymal transition, GBC: gallbladder cancer, GO: Gene Ontology, NF-kB: nuclear factor kappa B, PI3K-AKT: phosphoinositide 3-kinase-protein kinase B, PK/tox: pharmacokinetic and toxicological, PPI: protein-protein interaction.

To enhance the pharmacological value of network pharmacology and molecular docking in natural product-based research on GBC and BTCs, we propose a best-practice workflow centered on transparency, methodological rigor, and translational relevance (Figure 1).

Several groups have proposed general methodological frameworks for network pharmacology and related in silico analyses, particularly in the fields of traditional medicine and multi-component therapeutics [38,55,58]. These frameworks have made important contributions by emphasizing transparent reporting of compound lists, target prediction methods, network construction procedures, and experimental validation. Likewise, broader guidance on natural product research has highlighted the importance of reproducible intervention characterization, including source material, preparation, standardization, and analytical profiling [111]. In addition to these domain-specific considerations, we encourage researchers to align with established principles of computational research transparency, including clear documentation of code, software versions, and parameter settings; adherence to findable, accessible, interoperable, reusable (FAIR) data practices where feasible; and, for predictive models, TRIPOD-aligned reporting of model development, specification, and validation [112,113]. However, these recommendations are largely disease-agnostic and generally address cancer context, pharmacological prioritization, and downstream validation at a relatively broad level.

In contrast, our proposed workflow is tailored specifically to natural product-based interventions in GBC and BTCs and incorporates three features that are not typically foregrounded in existing frameworks. First, it embeds disease-specific target selection by requiring the explicit use, justification, and quality control of GBC/BTC-focused genomic and transcriptomic datasets, wherever feasible, when defining disease gene sets. Second, it introduces translational prioritization criteria that link network hubs and docking hits to achievable exposure levels, pharmacokinetic and toxicological plausibility, and realistic opportunities for combination with current GBC/BTC systemic therapies. Third, it formalizes an iterative cycle between in silico predictions and preclinical validation, with explicit attention to pharmacologically relevant readouts such as apoptosis, epithelial-mesenchymal transition, resistance-associated pathways, and chemosensitization endpoints in GBC/BTC models.

The workflow begins with the explicit definition of the pharmacological question and intervention. Authors should specify whether the objective is to elucidate mechanisms underlying observed anticancer activity, identify potential synergistic targets for standard chemotherapy, or prioritize compounds for further development in GBC and BTCs [114]. The natural product under investigation should be characterized as comprehensively as possible. This should include its composition, source, method of preparation, and any available pharmacokinetic or toxicological data, with the specific reporting elements summarized in Table 1.

**Table 1:** Minimal reporting checklist for network pharmacology and molecular docking studies of natural products in gallbladder and biliary tract cancers. BTC: biliary tract cancer, FGFR2: fibroblast growth factor receptor 2, GBC: gallbladder cancer, IDH1: isocitrate dehydrogenase 1, PDB IDs: protein data bank identifiers, PPI: protein-protein interaction.

Domain	Item to report	Key details/notes
Research question	Study objective and disease context	Mechanistic elucidation vs target prioritization vs combination design; specify GBC/BTC subtype, stage, and therapeutic context
Natural product characterization	Intervention description	Botanical/biological identity or source material; extraction/preparation method; standardization; analytical profile, where available; known pharmacokinetics/toxicity and traditional use
Compounds	Compound selection	Data sources (phytochemical profiling, curated databases); number of compounds; inclusion/exclusion criteria; rationale for filters (e.g., oral bioavailability, “drug‑likeness”)
Target prediction	Tools and parameters	Names of target prediction tools/databases; versions; species; score/threshold settings; how multiple tools were integrated or reconciled
Disease genes	Disease gene sources	Databases and omics datasets used to define GBC/BTC‑related genes; search terms; inclusion/exclusion criteria; any manual curation and quality control; explicit statement on whether GBC‑specific or BTC‑specific datasets were used and how they were integrated into the disease gene set
Network construction	PPI and network building	PPI database(s); interaction confidence thresholds; handling of isoforms; software/platform used; any filtering or simplification steps
Network analysis	Topological metrics and criteria	Which metrics were used (e.g., degree, betweenness, clustering coefficient) and why; thresholds for defining hubs, bottlenecks or modules
Functional/pathway enrichment	Enrichment methodology	Pathway/ontology databases; background gene set; statistical tests; approach to multiple‑testing correction; reporting of effect sizes and leading genes
Docking: structure selection	Protein and ligand preparation	PDB IDs and chains; isoform and conformational state choice; treatment of missing residues and post‑translational modifications; ligand preparation (protonation, tautomers, energy minimization)
Docking: protocol and validation	Docking procedure and interpretation	Docking software and key parameters; binding site definition; use of known ligands/controls; criteria for ranking; emphasis on comparative rather than absolute scores
Integration with experiments	Link to in vitro and in vivo data	How in silico predictions relate to existing experimental data for the same compounds and models; discrepancies and how they are interpreted
Translational planning	Proposed follow‑up and clinical relevance	Prioritized targets/pathways/compound–target pairs; proposed in vitro/in vivo assays and concentration ranges; potential combinations with standard therapies; pharmacokinetic, toxicity and drug–drug interaction considerations; description of how proposed targets and pathways relate to current gemcitabine–platinum regimens and, where relevant, to emerging BTC therapies such as FGFR2 and IDH1 inhibitors and immune checkpoint inhibitors (e.g. durvalumab, pembrolizumab); and whether network outputs suggest modulation of the tumor immune microenvironment (e.g. inflammatory cytokines, immune checkpoints, myeloid and lymphoid infiltrates) that could plausibly synergize with immunotherapy
Data and code availability	Transparency of inputs and analyses	Availability of compound lists, target sets, network files, and analysis scripts (e.g., as supplementary materials or in public repositories)
Rigor and transparency	Declaration of transparency and limitations	Completion of journal‑specific transparency/rigor checklists; explicit discussion of key methodological limitations and potential sources of bias

Next, compound and target selection should be both systematic and explicitly justified. For complex mixtures, comprehensive compound lists derived from analytical chemistry studies or well-curated databases should be used. Any filtering steps (e.g., based on oral bioavailability or “drug-likeness”) should be clearly justified in light of the anticipated route of administration and expected exposure [101,115]. Target prediction tools and disease gene resources should be selected with an awareness of their respective strengths and limitations. Key methodological choices and parameter settings, as outlined in Table 1, should be fully reported. Where feasible, disease gene sets should incorporate GBC- or BTC-specific transcriptomic and genomic data, together with transparent inclusion criteria and quality-control procedures [96,111,116].

When assembling networks and performing enrichment analyses, key parameters should be predefined and clearly reported. These include interaction confidence thresholds for protein-protein interaction networks, background gene lists, statistical tests, and methods for multiple-testing correction. In practice, we recommend the use of standard multiple-testing corrections such as the Benjamini-Hochberg false discovery rate (FDR) procedure, with clearly prespecified adjusted p-value or q-value thresholds (e.g., q < 0.05) for pathway and Gene Ontology enrichment analyses. Network visualizations should be accompanied by quantitative summaries and clear explanations of how topological metrics inform pharmacological hypotheses, rather than relying solely on visual complexity [117,118]. Enrichment results are best presented together with the underlying gene counts and effect sizes, rather than simply as lists of pathway names and p-values, allowing readers to assess their biological and pharmacological relevance more critically [66,119].

For molecular docking, protein structure selection and protocol validation are critical. Wherever possible, structures should be chosen to reflect biologically relevant isoforms, conformational states, and post-translational modifications. The rationale for these choices should be clearly stated. Binding sites should be defined using experimental evidence or robust prediction methods whenever available. Docking protocols should then be benchmarked against known ligands or reference compounds. Reporting should include docking scores, key predicted interactions, and recognized limitations. Emphasis should be placed on the comparative rather than absolute interpretation of predicted binding affinities [86,107,108,120-122].

Finally, each study should include a clear translational plan. This should involve identifying a limited number of high-priority targets, pathways, or compound-target pairs for experimental validation and outlining feasible in vitro and in vivo assays, together with pharmacologically relevant concentration ranges. It should also include consideration of how the findings might inform dose selection, treatment scheduling, or combination strategies with existing therapies in future pharmacological studies [55,123,124]. A concise reporting checklist, provided in Table 1, is intended to standardize practice, reduce the omission of critical methodological details, and facilitate critical appraisal by readers and reviewers.

Translational perspectives

When conducted rigorously and interpreted with appropriate caution, network pharmacology and molecular docking can serve as valuable components of an integrated pharmacological research program in GBC and other BTCs, particularly when viewed as exploratory tools rather than definitive sources of evidence [125]. By mapping the multi-target effects of natural products and aligning them with disease-specific pathways and mechanisms of drug resistance, these approaches can generate biologically grounded hypotheses [126]. They may help guide the selection of compounds, targets, and therapeutic combinations for subsequent mechanistic investigations. They can also identify potential toxicity liabilities and drug-drug interactions at an early stage of development [127]. Taken together, these contributions may support more rational hypothesis generation and pharmacological development, provided that limitations related to target prediction, network completeness, and docking accuracy are explicitly acknowledged and addressed.

For complex natural preparations, network-based analyses are particularly valuable during the hypothesis-generating stage. They can help disentangle the contributions of individual metabolites and highlight convergent mechanisms through which diverse constituents act on shared pharmacological nodes [57]. They may also suggest how these actions could interact with standard systemic therapies [128]. Crucially, however, in silico pharmacology should not be regarded as an endpoint or as a substitute for experimental evidence [129]. Its greatest impact will likely come when network and docking outputs are embedded within iterative cycles of experimental pharmacology, spanning cell-based assays, animal models, and early-phase clinical studies [130]. Within this framework, in silico methods generate and refine hypotheses, experimental findings feed back to update models, and clinical observations ultimately test the real-world relevance of proposed mechanisms [131].

Looking ahead, network pharmacology for natural products is increasingly being enhanced by AI-assisted approaches. Recent studies have combined machine learning and deep learning with target prediction, virtual screening, and docking workflows to prioritize multi-target drug candidates and address the chemical diversity of complex natural product libraries [132,133]. Such approaches may be particularly valuable in GBC/BTC, where relatively small and heterogeneous datasets could benefit from integrative modelling across multiple omics layers and pharmacological phenotypes.

Limitations of this review

This is a narrative rather than a systematic review. The literature search was restricted to selected bibliographic databases and peer-reviewed English-language publications; therefore, relevant studies published in other languages or contained within the grey literature may have been missed. The review focused primarily on original reports involving network pharmacology, molecular docking, and related experimental research. Existing systematic reviews and meta-analyses were used principally to provide context rather than as sources for exhaustive evidence synthesis. The included studies were heterogeneous with respect to natural product characterization, network construction, docking protocols, and experimental validation, limiting direct comparison across methodologies. In addition, the methodological recommendations proposed here are based on critical synthesis and interpretation of a diverse body of literature rather than on formal consensus-building approaches. These considerations should be borne in mind when interpreting both the strengths and the limitations of the workflow and reporting checklist proposed in this review.

## Conclusions

In summary, natural product-based therapies remain a promising yet underdeveloped pharmacological area in GBC and BTCs. Network pharmacology and molecular docking provide a potentially useful framework for exploring multi-target actions and for organizing complex preclinical findings into mechanistic hypotheses. However, their outputs should be regarded as hypothesis-generating rather than confirmatory, and their translational value depends heavily on transparent reporting, biologically grounded disease modeling, and rigorous experimental validation. Adoption of best-practice workflows and clearer reporting standards may improve the interpretability and pharmacological relevance of future studies in this area. In that setting, these approaches may help support more mechanism-informed preclinical development of complex natural products and phytochemicals in hepatobiliary oncology.
